# The virtual brain: 30 years of video-game play and cognitive abilities

**DOI:** 10.3389/fpsyg.2013.00629

**Published:** 2013-09-13

**Authors:** Andrew J. Latham, Lucy L. M. Patston, Lynette J. Tippett

**Affiliations:** ^1^School of Psychology, The University of AucklandAuckland, New Zealand; ^2^Centre for Brain Research, The University of AucklandAuckland, New Zealand

**Keywords:** video games, expertise, cognitive training, transfer of training, perceptual learning

## Abstract

Forty years have passed since video-games were first made widely available to the public and subsequently playing games has become a favorite past-time for many. Players continuously engage with dynamic visual displays with success contingent on the time-pressured deployment, and flexible allocation, of attention as well as precise bimanual movements. Evidence to date suggests that both brief and extensive exposure to video-game play can result in a broad range of enhancements to various cognitive faculties that generalize beyond the original context. Despite promise, video-game research is host to a number of methodological issues that require addressing before progress can be made in this area. Here an effort is made to consolidate the past 30 years of literature examining the effects of video-game play on cognitive faculties and, more recently, neural systems. Future work is required to identify the mechanism that allows the act of video-game play to generate such a broad range of generalized enhancements.

Video-game play has become the past-time of choice for current generations, with entertainment software allowing individuals to engage both socially and competitively with individuals across the globe. Players engage with seamless virtual environments with success contingent on the execution of precise bimanual motor movements in response to complex visual cues. Evidence suggests that extensive video-game play may lead to the enhancement of visual attention and executive control, generalizing beyond the context of the video game, although this is still debated.

Video games became publically available for the first time in 1972 with the release of the first arcade machine, and household gaming console, the *Magnavox Odyssey*. While this new technology was quickly seized upon by the public, it was not without objection. Many saw video-game play as a mindless exercise imparting no real benefit to the player. Ball (1978) defended video-game play and suggested it offered a potential mechanism to improve a variety of cognitive skills including eye-hand coordination, decision making, following directions, and number and word recognition. Many publications that address these claims have followed. In this paper we present a comprehensive review of studies over the last 30 years that contribute both to current views concerning the effects of video-game play on specific cognitive domains and to their neural underpinnings.

This review is organized primarily by cognitive functions that are argued to be influenced by video-game play. It also examines the small literature addressing the neural underpinnings of superior behavioral performance in video-game players, and considers the challenges to, and limitations of, the video-game literature. Results of all the studies reviewed are summarized in Supplementary Table 1.

## Hand-eye coordination and reaction time

Griffith and colleagues published the first finding demonstrating individuals with video-game experience outperforming those with no video-game experience. They reported superior performance by VGPs on a rotary pursuit task, suggesting they possessed enhanced hand-eye coordination (Griffith et al., 1983). Despite this striking result, there was no relationship between participants' rotary pursuit performance and the intensity of their video-game play (hours per week) or length of exposure to video-game play.

Early studies also indicated that video-game play could enhance visuomotor reaction times to a wide range of stimuli. Orosy-Fildes and Allan (1989) tested participants on a simple color discrimination task and found that participants who played a video-game on the Atari 2600 system responded significantly faster than those who did not play video games. This difference may also, however, result from a greater capacity to perform visual discriminations, or from the effects of greater cognitive stimulation. Similarly faster reaction times were observed in simple color and shape discrimination tasks by children classified as video-game players (VGPs) according to their enthusiasm toward video-games, compared to non-video game players (NVGPs) who were not enthusiastic toward video games (Yuji, 1996). Of course children who possess faster reaction times may be more enthusiastic toward video-games because of the advantage their innate quickness may afford them during video-game play. Nevertheless, faster reaction times in response to visual stimuli by expert VGPs, and by NVGPs after video-game training, is a recurrent finding throughout most of the video-game literature (e.g., Castel et al., 2005; Bialystok, 2006).

## Spatial visualization

One of the first ideas to receive close examination was that video-game play might increase proficiency at spatial processing, particularly spatial visualization. Gagnon (1985) assessed the impact of video-game training on performance on the Guildford-Zimmerman spatial visualization and spatial orientation test, along with visual pursuit, manual speed and manual accuracy subtests from the Employee Aptitude Test. Only female participants showed significantly improved post-play performance, and even then only for spatial visualization, but interestingly the amount of prior video-game experience that participants possessed correlated significantly with visual pursuit, spatial visualization, and hand-eye coordination performance. Controlling for prior video-game experience, Dorval and Peìpin (1986) assessed the impact of video-game training on the spatial relations portion of the Differential Aptitude test relative to controls who refrained from video-game play. Both males and females who underwent video-game training showed significantly enhanced performance on the spatial relations subtest. However, due to the lack of an adequate control condition in this experiment, and in many studies to follow, we cannot rule out the possibility that general cognitive stimulation may have improved performance.

Similar improvements in spatial visualization after video-game training have also been observed in children (Subrahmanyam and Greenfield, 1994). This study is particularly note-worthy as it was the first to observe the effects of video-game genre on the outcomes of video-game training. Only children who played the spatially demanding video-game, *Marble Madness*, showed significantly improved spatial visualization performance, while children who played the language-oriented video-game, *Conjecture*, showed none. The use of another video-game as a control went some way toward adjusting for the effects of general cognitive stimulation, although it remains possible that there is a greater expectancy that spatial performance will improve after playing spatially demanding games (*Marble Madness*) than after playing non-spatial games (*Conjecture*), which may account for at least part of the result (Boot et al., 2011).

Many early studies examined the link between video-game training and mental rotation performance, a specific form of spatial visualization. McClurg and Chaillè (1987) found improvements on mental rotation performance in children after video-game play; however, this study lacked an adequate control condition. Okagaki and Frensch (1994) found that video-game naïve participants who played the spatially-oriented video-game *Tetris,* showed improved performance on paper-and-pencil measures of mental rotation and spatial visualization relative to participants who did not play *Tetris*. Significantly, this is one of the only studies to show that expertise acquired during video-game play may generalize beyond performance in computerized environments to non-computerized performance, which provides a stronger test of the transfer of the proficiency (Boot et al., 2011). Unfortunately, Okagaki and Frensch did not include an adequate control condition.

Not all findings involving spatial video-game play (*Tetris*) and mental rotation performance have been consistent. Sims and Mayer (2002) compared the mental rotation performance of skilled and non-skilled *Tetris* players, and also compared mental rotation performance of non-skilled *Tetris* players before and after training. Skilled *Tetris* players outperformed non-skilled *Tetris* players only when stimuli were identical or similar to those within *Tetris*. Furthermore, no performance gains were observed on mental rotation in *Tetris*-trained participants compared to those who received no training. In contrast to Okagaki and Frensch (1994), this study suggests superior mental rotation performance may not generalize beyond the types of stimuli video-game players are trained upon. In summary, simple video-games such as *Tetris* and *Blockout,* may convey a limited enhancement as success in these games is primarily reliant on mental rotation skill with a limited range of stimuli.

There has been particular interest in the link between spatially-oriented video-games and the reduction of gender disparities in mental rotation performance, although close examination of the findings reveals mixed outcomes. Although De Lisi and Cammarano (1996) concluded that the expected gender difference in favor of men was attenuated in participants who played *Blockout,* they also noted the direction of this disparity was actually reversed in their control group who played only the non-spatial game *Solitaire*. Indeed the magnitude of improvement and absolute scores of the females groups playing the two types of computer games was comparable. This is far from convincing evidence that playing spatially-oriented video-games (in this case *Blockout)* differentially improves the performance of females on mental rotation. More convincing changes were observed, however, in a study with children (De Lisi and Wolford, 2002). Pre-training gender differences on a mental rotation task disappeared following video-game training (on either *Tetris* or the non-spatial game *Carmen Sandiago*), largely due to the extent of the improved performance of the girls who played *Tetris*. Overall children who played *Tetris* improved significantly in comparison to controls who played *Carmen Sandiago*. While these results may be driven simply by the expectation to improve in mental rotation performance after playing *Tetris,* this does not account for the differential improvements of the girls who play *Tetris.*

## Visuospatial attention

The largest body of video-game literature examines the impact of video-game play on the capacity and spatial distribution of visuospatial attention. Success in many action video-games requires participants to attend to disparate multiple in-game objects all within complex visual environments. Given these demand characteristics, it is perhaps not surprising that most studies report enhancements in these abilities in skilled VGPs.

Greenfield et al. (1994) began this wave of research, when they assessed the impact of video-game expertise and training on a task that requires dividing visual attention. Participants indicated whether a flash of light occurred on the left or right of fixation, or at both locations concurrently. Presentation probabilities were fixed so that in one condition the target was presented 10% of the time to one location, 80% of the time to the other location and 10% of the time to both locations concurrently, while in the other condition the target appeared 45% of the time in each position and 10% of the time to both locations concurrently. Both NVGPs and expert VGPs showed a reaction time benefit to high probability locations, however, expert VGPs, unlike the NVGPs, did not show an attentional cost at the low probability position. This suggests that expert VGPs are superior at dividing visual attention. This result could also be explained by a larger attentional window or a larger distribution of spatial attention. A follow-up training study showed that NVGPs who played *Robotron: 2084* for 5 h, showed results in the same direction as expert VGPs. Unfortunately, there was no control training condition.

Current interest in the effects of *action* video-game play on visual attention has largely been stimulated by the seminal work of Green and Bavelier (2003), who compared expert VGPs and NVGPs on a battery of visuospatial and attentional tasks. A flanker compatibility task and enumeration task were used to measure attentional capacity. In the flanker compatibility task participants had to indicate which of two targets appeared in one of six possible locations while ignoring an outside distracter; in the enumeration task, participants had to indicate as quickly as possible the number of briefly flashed items that appeared. A useful field of view (UFOV) task was used to measure the spatial distribution of the field of attention. Participants had to locate on which radiating spoke a target was presented at up to 30° eccentricity. Finally an attentional blink task was used to test temporal aspects of visual attention. Participants were asked to identify a first target and to detect a second target that appears within a few hundreds of milliseconds later. Expert VGPs outperformed NVGPs on all tasks, suggesting they may possess a larger attentional capacity, larger field of attention and reduced attentional blink. Subsequently, Green and Bavelier (2006a,b) replicated and elaborated on these findings. Using a flanker task and manipulating perceptual loads, they found expert VGPs had greater attentional resources and distribution of visual attention, (Green and Bavelier, 2006a). In the same study they also replicated their UFOV findings of enhanced spatial distribution of attention across a wide field of view in VGPs, even when performing an additional central discrimination task. This indicates that superior UFOV performance was not due to the redistribution of attentional resources from central to peripheral vision. A training study showed these enhancements could be brought about in NVGPs after a small period of action video-game play (*Unreal Tournament 2004*) but not after playing a non-action video-game (*Tetris*).

Using an enumeration task Green and Bavelier (2006b) showed expert VGPs can enumerate about two more items than NVGPs and additionally possess superior performance on a multiple object tracking task. A training study suggested a causal role for action video-game play, as NVGPs trained on an action video-game (but not those trained on a non-action video game), showed similar enhancements. The authors argue these findings may be mediated by changes in visual short-term memory skills. Consistent with this, Boot et al. (2008) found VGPs showed superior multiple object-tracking performance and, while they did not find a significant different on an enumeration task, they did find expert VGPs performed significant better on a visual short-term memory task. This task involved detecting changes in a display comprising varying color lines at one of four orientations, which reappeared following a delay of 900 ms. Sungur and Boduroglu (2012) also reported that expert VGPs could more accurately track multiple objects and additionally showed a smaller performance deficit when required to hold the unique identities of multiple target elements whilst simultaneously tracking them. They also suggested this reflects larger visual short-term memory capacities of VGPs.

Many of the performance advantages shown by Green and colleagues in adult expert VGPs have been replicated in child and adolescent VGPs. Dye et al. (2009) found both child and adult VGPs displayed a flanker compatibility effect using the attentional network task. Similarly, Dye and Bavelier (2010) found both child and adult expert VGPs showed enhanced performance on the UFOV (with an additional central discrimination task), attentional blink, and on a multiple object tracking task. Unfortunately, training studies with children were not possible as the majority of action video-games are restricted for mature or adult audiences. It is possible, of course that children and adolescents who possess pre-existing superior visuospatial and attentional abilities are drawn to action video-games because these abilities result in greater success playing video games, and consequently playing games is more rewarding.

## Visual anticipation and visual search strategies

Kuhlman and Beitel (1991) found that child VGPs had a greater ability to anticipate stimulus onset. Anticipation is approximated by measuring the accuracy of participant responses to the timing and final location of stimuli in cases of apparent motion (e.g., Haywood, 1977). Child VGPs responded significantly more accurately to the final light stimulus in a sequence, suggesting they had better anticipated its arrival. Enhanced priming of stimulus presentation in expert VGPs, both in terms of location and content, may facilitate anticipation of the stimulus' arrival, resulting in faster responses once stimuli are presented. It remains possible, of course, that enhanced anticipation is not the result of video-game play but a pre-existing characteristic of those children who become VGPs.

Whether anticipatory abilities are related to visual search strategies is a matter of debate. Within the VGP literature, evidence related to enhanced visual search is mixed. Castel et al. (2005) investigated the effect of extensive video game play on visual search capability and inhibition of return. While expert male VGPs showed significantly faster reaction times on both the “easy” and “hard” visual searches and the inhibition-of-return task, contrary to expectations, no differential suppression of previous search locations was found. A similar pattern of simple reaction time benefits has also been reported by Bialystok (2006) who found that while expert VGPs made significantly faster responses than NVGPs on the Simon task, they did not show any difference in the effect of stimulus-response in/congruency. These simple reaction time benefits suggest that at least some of the task benefits shown by expert VGPs may simply be due to a heightened ability to make appropriate and rapid motor responses to visual stimuli.

Contrary to Castel et al. (2005), Hubert-Wallander et al. (2011) provided evidence that expert VGPs were capable of quicker visual searches during their “hard” visual search condition. Clark et al. (2011) suggested that expert VGPs may possess altered top-down search strategies, with the use of broader search patterns. In a modified change detection paradigm, expert VGPs not only correctly located changes more quickly than NVGPs, but appeared to do so as a result of a more efficient search strategy. This was shown by a tendency to mentally travel larger visual distances between change localization attempts, distribute and make change localization attempts more often in all four screen quadrants, and cover a larger visual area with change localization attempts in the first five attempts. Training studies are needed in order to determine whether faster visual searches and broader top-down search strategies result from video-game play or are a pre-existing characteristic of expert VGPs.

## Temporal dynamics of sensory attention

West et al. (2008) compared the performance of matched male expert VGPs and NVGPs on a temporal order judgment task that measures participants' sensitivity to an exogenous cue to a spatial location. Participants were presented two target items separated by a variable stimulus onset asynchrony and asked to report which target appeared first, with the location of one of the stimuli cued. The dependent measure is the point of subjective simultaneity, which indicates the stimulus onset asynchrony needed for a participant to perceive both targets as occurring simultaneously. Surprisingly, the point of subjective simultaneity in expert VGPs occurred at a significantly larger stimulus onset asynchrony than for NVGPs, suggesting VGPs attention may have been captured more by the exogenous cue (see section Exogenous and Endogenous Attention). Donohue et al. (2010) found that when no exogenous cue was present, expert VGPs were significantly more accurate than NVGPs at discriminating presentation asynchronies in visual and auditory stimuli. Incredibly, expert VGPs showed a point of subjective simultaneity that did not differ from simultaneous stimulus onset. Consistent with this, on judgments of multisensory temporal order when the stimulus asynchrony was 0 ms, NVGPs showed a response bias toward an earlier visual stimulus onset while expert VGPs were at chance, indicating no bias toward either modality. Donohue and colleagues also found a significant correlation between video-game experience and point of subjective simultaneity, suggesting better multisensory precision may result from increased video-game experience, although it is possible that individuals with better multisensory precision play more video-games due to the advantage this skill conveys.

Li et al. (2010) showed a reduced effect of backward masking in expert VGPs. In a follow-up training study only those NVGPs who played an action video-game (and not a non-action video-game, *The Sims*), showed a significant reduction in backward masking in the same direction as expert VGPs. However, this result could be driven by the expectation of NVGPs trained on the action video-games to improve on backwards masking, especially as the temporal demands of *The Sims* are low, because players able to control the speed of in-game time and perform actions while the game is paused.

## Exogenous and endogenous attention

The impact of exogenous cues on the performance of expert VGPs was further examined by West et al. (2008) using a signal detection paradigm. Participants viewed a UVOV display with three ringed eccentricities and populated with “swimmers” in constant motion; the swimmers were depicted by a circle with two “arms” represented by perpendicular lines. Participants responded whenever a swimmer began to struggle (move erratically). Expert VGPs showed significantly faster reaction times to erratic swimmers in all but the highest stimulus load and eccentricity, suggesting an increased sensitivity to changes in salient motion. Hubert-Wallander et al. (2011) also tested the impact of exogenous cuing but on a modified Posner cuing paradigm. Participants indicated whether a “T” presented to one of four target locations was upright or upside down. Each trial was preceded by an uninformative exogenous cue to one of the target locations. While expert VGPs identified the correct orientation significantly more quickly, there was no significant difference between expert VGPs and NVGPs in reaction time costs generated by the exogenous cue. Contrary to West et al. (2008), the authors suggested that video-game play may preferentially target endogenous as opposed to exogenous attention. It may simply be the case, however, that if expert VGPs know a cue is always a distracter, they are not preferentially captured by the exogenous cuing.

Chisholm et al. (2010) provided support for the view that expert VGPs possess enhanced endogenous attention. On an attentional capture task, participants identified the orientation of a line in a target element, either in the presence or absence of distractor information. Expert VGPs not only correctly responded significantly more quickly, regardless of whether distractors were present or not, but were also less adversely affected by distractor presence than NVGPs. The authors suggested expert VGPs may possess greater control of endogenous attention enabling them to suppress or disregard irrelevant information more easily than NVGPs. However, this result could also simply arise from reduced sensitivity to distractors without impacting on endogenous attention.

## Task switching

Video-game play has also been found to impact on cognitive task set selection and the higher-order capacity to switch between tasks. For example, Nelson and Strachan (2009) found participants' performance on a target location and matching figures task differed according to whether they had played the action video-game *Unreal Tournament* or puzzle video-game *Portal*. Participants who played the action video-game showed faster response times but lower accuracy, while participants who played the puzzle video-game showed slower response times but greater accuracy. This provided evidence that cognitive strategies primed by the demands of different video-game genres could possibly generalize beyond the context of the video-game itself.

Colzato et al. (2010) investigated the effect of action video-game expertise on task switching. Expert VGPs showed a significantly lower task switching cost than NVGPs when switching between identifying local and global cues. Karle et al. (2010) investigated what may underlie lower task switching costs in expert VGPs. They speculated that task switching is a combination process and lower costs may result from a greater capacity to activate relevant task networks, inhibit previously active and competing task networks, or a combination of both. Expert VGPs showed reduced task switching costs relative to NVGPs in non-overlapping tasks (i.e., character discrimination and numeric discrimination) but showed no significant reduction in task switching costs when tasks overlapped (i.e., odd/even, prime/multiple, less than/more than). The authors argued that overlapping tasks create proactive interference that requires inhibiting conflicting response sets, which suggests that lower task switching costs shown by expert VGPs may reflect an enhanced ability to activate task relevant networks.

## Fundamental properties of the visual system, visual perception and use of sensory evidence

Some of the most exciting, diverse and novel findings in the VGP literature have concerned the potential enhancement of fundamental aspects of the visual system. Green and Bavelier (2007) compared the performance of male expert VGPs and matched NVGPs on a visual crowding paradigm. Participants were presented with three Ts randomly oriented either the right way up or upside down at three different eccentricities, and had to identify the center T's orientation. The effect of visual crowding was modulated by moving the Ts closer together or further apart. Expert VGPs not only show a reduced effect of crowding but were able discriminate the orientation of significantly smaller Ts than NVGPs, indicating they may possess greater visual acuity. A follow-up training study produced mixed results with NVGPs trained on an action video-game showing improved visual crowding performance but not visual acuity. NVGPs trained on the non-action video-game (*Tetris*) showed no improvement. The inability to produce enhanced visual acuity in NVGPs may mean it is not amenable to short-term training, or instead that it represents a pre-existing characteristic of expert VGPs.

Contrast sensitivity, another fundamental property of vision, has also been found to be superior in expert VGPs. Contrast sensitivity refers to an individual's ability to detect small increments in shades of gray on a uniform background, and like visual acuity, it is a major limiting factor in determining quality of sight (Campbell, 1983). Li et al. (2009) found expert male VGPs were more accurate than NVGPs on a standard contrast sensitivity paradigm. In a follow-up training study, NVGPs who played action video-games significantly improved their contrast sensitivity while NVGPs who trained on the non-action video-game (*The Sims 2*) showed no improvement.

In addition to increased quality of vision, video-game expertise is also associated with larger central and peripheral visual fields, determined using Goldmann kinetic perimetry (Buckley et al., 2010). During this technique, participants respond once they detect a circular light target (328 candelas for peripheral field and 20 candelas central field) that traverses from 100° eccentricity to central fixation along a straight line. Eccentricities where target detection occurred were recorded and the task repeated until a full visual field boundary in 30° steps was constructed for each participant. Amazingly, expert VGPs showed central and peripheral visual fields that were around 1000 deg^2^larger (around 50% larger central visual field and 15% larger peripheral visual field, respectively) than NVGPs.

At a higher level of the visual system, Sungur and Boduroglu (2012) investigated the effects of video-game expertise on the resolution of visual representations. On the color wheel task, expert VGPs were able to identify the color of a target on a color wheel with 180 options more precisely that NVGPs. Sugur and Boduroglu also calculated the magnitude of the errors on both the color wheel and a UFOV task. Expert VGPs made a greater proportion of small magnitude errors (i.e., a few degrees on the color wheel or neighboring items on the UFOV) than NVGPs, while NVGPs made a higher proportion of high magnitude errors (i.e., guesses) than expert VGPs. The authors argued that this showed that visual information was stored at a higher resolution by expert VGPs. It is possible of course, that the higher proportion of “guesses” made by NVGPs may reflect failures to detect targets rather than lower resolution representations.

Finally, Green et al. (2010) provided compelling evidence that VGPs make more efficient use of sensory evidence in making judgments. They applied a drift diffusion model to participant's responses on a visual motion direction discrimination task, in which they judged the net movement of a dot array under different levels of dot movement coherency. VGPs accumulated and integrated visual sensory information at a faster rate than NVGPs, enabling more rapid inferences to be made, particularly at lower coherence. The same results were observed in an auditory tone location discrimination task suggesting they reflect a multi-modal enhancement in sensitivity in VGPs. The results of a follow-up training study supported the link with action video-game play in the visual domain, with nearly identical results to those of experienced VGPs; NVGPs trained on an action video-game showed significantly better performance than NVGPs trained on a non-action video-game (*The Sims 2*), who showed no improvement.

## Video-game play as a cognitive intervention

Video-game play was first tested as an intervention to stem cognitive decline in older adults in the mid-1980s. Early results showed promise, with improved performance of older adults on a range of cognitive measures (e.g., WAIS-R, Sternberg paradigm), following video-game play (e.g., Drew and Waters, 1986; Clark et al., 1987; Dustman et al., 1992; Goldstein et al., 1997). These studies failed, however, to include an adequate control condition (if one was included at all), limiting interpretation.

More recently, Basak et al. (2008) tested the impact of playing the action video-game *Rise of Nations* on older adults. Unlike typical action games tested in the video-game literature, *Rise of Nations* is a real-time strategy game. Players control a nation's economy, military, technological development, and diplomatic relations with neighboring societies. Success can be achieved either through military dominance, cultural dominance, or constructing a wonder of the world. Older adults in the video-game play group showed significant improvements in working memory, abstract reasoning, distractor inhibition, mental rotation performance, and a significant reduction in task-switching costs relative to older adults who did not play. Unfortunately, like earlier intervention-based studies, the inadequate control condition means the significant improvements may have resulted from greater cognitive stimulation.

## Neuroimaging and video-game play

Evidence from studies involving video-game training suggest that video-game play, even for a short period of time, may result in enhancement to a large number of cognitive abilities that last for at least 4 months (e.g., Li et al., 2010). The neural correlates underpinning these superior abilities are not well-understood as only a handful of neuroimaging studies have addressed this question.

Haier et al. (1992) conducted the pioneering neuroimaging study of video-game play. Eight participants underwent positron emission tomography (PET) scanning while playing the non-action video-game *Tetris* both before, and after, daily practice for 4–8 weeks. In addition, 16 control participants were scanned while passively viewing visual stimuli. Participants who underwent the video-game training showed a significant decrease in whole brain glucose metabolism. Interestingly, the level of improvement shown by participants in *Tetris* was inversely correlated with levels of glucose metabolism. Haier and colleagues suggest that the decrease in glucose metabolism may reflect a more efficient utilization of the neural circuitry brought about through learning and task mastery. While this may explain a portion of the decrease to whole brain metabolism, it does not explain why whole brain glucose metabolism was lowered as the enhancements facilitated by *Tetris* are likely restricted to areas subtending mental rotation performance only (see earlier discussion in section Spatial Visualization).

Granek et al. (2010) used event-related fMRI to investigate the effect of video-game expertise on cortical activity during the preparation of visually-guided movements. During scanning participants completed four visuomotor tasks. These involved navigating their hand or a cursor control via a joystick toward, or away from, a fixated target. Visuomotor planning typically comprises a network of prefrontal, premotor, sensorimotor, and parietal regions (Gorbet et al., 2004; Gorbet and Sergio, 2007). Expert VGPs and NVGPs appeared to show dissociation in activation weightings in this network with VGPs showing greater prefrontal activation and NVGPs showing greater parietal activation. The authors suggest that these differences may reflect differences in the familiarity of expert VGPs and NVGPs with novel visuomotor tasks. Expert VGPs may not have to attend as closely to the outcomes of their motor acts as they are able to generalize visuomotor response patterns developed through video-game play. Conversely, NVGPs may have to attend more closely to the outcomes of their actions as they have not yet developed the appropriate response patterns. A future training study is required to show these hemodynamic response patterns to motor preparation are the result of video-game play and not a pre-existing characteristic of expert VGPs.

Mishra et al. (2011) compared expert VGPs and matched NVGPs on a selective attention task during recording of 62-channel EEG. During the experiment participants were required to respond to all instances of a numeral presentation in one cued letter stream, while ignoring those occurring in distractor streams. Of particular interest was the steady state visual evoked potential (SSVEP), which is a frequency-tagged neural response to periodic stimuli. When attention is directed at the periodic stimuli a larger SSVEP amplitude is generated. While no significant group differences were found in the SSVEP amplitude for attended stimuli, expert VGPs did show significantly reduced SSVEP amplitudes for unattended stimuli. Consistent with the behavioral findings of Chisholm et al. (2010), this suggests that enhanced attentional performance in expert VGPs may be underpinned by a greater capacity to suppress, or disregard, irrelevant stimuli. Interestingly, expert VGPs also showed larger P300 amplitudes relative to NVGPs in response to numerical targets under high load conditions. This suggests that expert VGPs may show greater sensitivity than NVGPs to task-relevant stimuli under increased load, which in turn may underpin their more accurate behavioral performance.

Bavelier et al. (2012) compared activation of expert VGPs and NVGPs in the fronto-parietal attentional network and the motion sensitive area (MT/MTS) during a visual search task with distractors. Participants had to indicate whether a diamond or square was present in a ring of shapes surrounding the central fixation cross, performed under either a low or high stimulus load. This was accompanied by distractors—a static or moving patch of dots presented centrally or peripherally to either side of fixation. NVGPs, unlike expert VGPs, showed a significant increase in activation of the fronto-parietal attentional network in response to increased visual search load. Bavelier and colleagues suggested the heavily reduced utilization of this network in expert VGPs, may reflect lower use of attentional resources with higher task burdens in contrast to NVGPs. Additionally, moving distracters (patches of irrelevant moving dots) elicited less activation in MT/MST in expert VGPs than NVGPs but not in response to attended motion stimuli. Consistent with previous research (Chisholm et al., 2010; Mishra et al., 2011) this finding suggests that VGPs are more efficient at suppressing, or disregarding, irrelevant stimuli.

To date the small neuroimaging literature on expert VGPs has focused on the functional correlates of enhanced cognitive performance. Substantial evidence in the broader neuroimaging literature indicates that practice and expertise can induce not only functional but structural changes to neural organization. For example, repetition of simple finger exercises has been shown to lead to increased cortical representation of the fingers (Pascual-Leone et al., 1995). Expertise has also been shown to be accompanied by structural changes in brain regions and pathways associated with that expertise (e.g., taxi drivers, Maguire et al., 2000; bilingual individuals, Mechelli et al., 2004). Expert musicians have provided a model for the investigation of neuroplasticity and there is now considerable evidence that the brains of adult musicians differ from those of non-musicians both functionally and anatomically (Jäncke, 2009; Oechslin et al., 2010; Wan and Schlaug, 2010). Future neuro-imaging research in VGPs should also investigate structural changes that may accompany video-game expertise or training, as well as addressing the question of whether extensive training can shape the neural representation of cognitive processes *beyond* specific trained skills.

## Challenges to the video-game play literature

Not all findings in the video-game literature have been consistent or successful. For example, while Boot et al. (2008) found expert VGPs showed superior visual short term memory, multiple object-tracking, mental-rotation performance and reduced task switching costs, they found no improvement on block tapping, enumeration, functional field of view, operations span, Ravens Matrices, spatial 2-back, Tower of London, and no reduction in attentional blink. Follow-up training by NVGPs on an action video-game also failed to elicit any enhancement. Direct replication attempts of Green and Bavelier's (2003) seminal findings have also been mixed. Irons et al. (2011) failed to replicate the flanker compatibility effect while Murphy and Spencer (2009) failed to replicate any of the original findings at all.

Boot et al. (2011) have argued that there are a variety of methodological limitations within the video-game literature, many of which have been highlighted throughout the current review. Specifically, they list the use of either unspecified, or overt recruiting methods, which they argue potentially prime the VGP stereotype and expectancy effects in VGP participants; the inability to infer directional effects from cross-section research designs; the similarity between the experimental task (generalization task) and video-game experience and the possibility of placebo effects in training studies. Indeed these types of problems are ubiquitous in most investigations of expertise with specialist populations. Researchers investigating video-game play and expertise can take the lead by employing clinical trial best practices (Boot et al., 2011) to clarify results in what appears to be an exciting avenue of research.

Four additional limitations appear particularly salient in the video-game literature to date. First, the majority of researchers have employed computerized experimental paradigms that typically require rapid target detection with manipulations to distractor difficulty and target eccentricity. Arguably, these tap the same skills in a very similar environment to the “training environment” (i.e., video-game play) and as a result may not provide evidence of proficiency beyond training. Exceptions showing the generalization of expertise or training to non-computerized tasks do exist (e.g., Okagaki and Frensch, 1994), but are rare. If at all possible, researchers should include non-computerized transfer tasks.

Second, there are large disparities in the relative frequency of males and females within VGP and non-VGP populations (with the former predominantly male and later predominantly female). Quaiser-Pohl et al. (2006) examined the effect of video-game genre preference on mental-rotation performance and gender differences. Latent class analysis on 861 adolescence revealed that males were significantly over-represented in the action video-game category (81.7%), while females were over-represented in the puzzle video-game (82.9%) and non-VGP (81.9%) categories. It cannot be ruled out that the greater representation of expert male VGPs is due to self-selection. Spence et al. (2009) directly investigated the impact of gender by comparing the attentional performance of male and female NVGPs, who were matched by their baseline score, after video-game training. Reassuringly, both males and females showed the same systematic enhancement to attentional performance. Interestingly, follow-up testing at four months showed that these performance gains remained even when video game play had ceased. Similar long-term enhancements have also been found by Feng et al. (2007) on UFOV and mental rotation performance 5 months after video-game play had ceased.

Third, there are difficulties in adequately defining and measuring video-game expertise. Cross-sectional studies often classify video-game players as “experts” if they have played video-games for a small period of time per week over the past 6 months. Unfortunately, there is no guarantee that individuals with these video-gaming characteristics possess expertise, or could even obtain expertise with such a small time commitment. Greenfield et al. (1994) were the first researchers to use a predefined group of “expert” VGPs to assess the effects of prolonged video-game play. Expert VGPs were participants who were able to reach a score of over 200,000 on the action video game Robot Battle, while video game novices were participants who were unable to reach a score of 20,000. Unfortunately there is no standardized objective measure of performance expertise for video-game play, unlike some expert populations (e.g., “Grades” in musical performance and theory). The lack of such a metric makes it difficult to assess performance not only within a video-game genre but also between genres. Certain popular game titles, however, currently have, or are beginning to utilize, skill metrics to match players of comparable skill in multiplayer games. For example, the most recent release of *Counter Strike: Global Offensive* and popular multiplayer online battle area *League of Legends* use performance ratings. *Starcraft II* uses a total of seven leagues ranging from the beginners “Bronze” league to the “Grand Master League” which consists of the top 200 players on a server. With the rise of professional video-game play, level of professional attainment (i.e., as a national or international competitor) could also serve as a proxy for expertise. While not perfect, future studies may be able to tap into skill metrics, online rankings, and level of professional attainment in order to better assess the expertise of their expert VGP samples.

Lastly, more emphasis is needed on the links between the specific game genres of expert VGPs, video-game training and the cognitive enhancements measured. Throughout this review it is evident the findings of earlier studies (during the 1980's and early 1990's) are typically weaker and less precise than those of more recent studies. While this often reflects study designs, findings were also shaped by the video-games of that era, which were generally extremely simple in nature, requiring only repetitive and distinct patterns of responses to succeed. Overall it appears that training on this sort of game can lead to improved performance, but improvements are generally are restricted to the context in which training occurs and reflect increased task efficacy, rather than more general enhancement of underlying cognitive processes (Ball et al., 2002). As information technology progressed (both software and hardware) so too have the cognitive demands of the plethora of action video games played today. Although the term “action video game” is typically used in the literature to refer simply to “first person shooters,” where success is heavily reliant on the ability to make speeded responses to the visual stimuli present on the screen at any given moment, “action video games” of today in fact encompass a vast array of different game genres (e.g., first-person shooter, real-time strategy, role-playing game, multiplayer-online battle arena, etc.) each with the potential to give rise to a unique set of perceptual and cognitive enhancements. In contrast to “first person shooter” games, success in real-time strategy games, for example, is more reliant on the ability to continually assess, update, and plan the most efficient series of responses to visual stimuli and as a result may preferentially enhance more executive processes. Furthermore, objects located in modern video games are no longer just passive; through the integration of artificial intelligence and multiplayer capabilities they are able to learn and adjust to the player as the game progresses. Success against “others” requires the recruitment of additional higher order cognitive processes such as theory of mind (Premack and Woodruff, 1978) in order to infer and attribute knowledge, perception and false belief states to dynamically-controlled in-game items. While there is undoubtedly overlap between the skill sets required by different game genres, the specific enhancements they drive are likely to closely reflect the cognitive domains they actively recruit (e.g., Basak et al., 2008; Nelson and Strachan, 2009; Li et al., 2010). Future studies would benefit from careful analysis of cognitive demands of the specific game genres of expert VGPs, and the cognitive domains assessed when testing for generalization effects.

## Conclusion and future directions

Evidence is continuing to accumulate that suggests both brief and extensive experience with video-games can result in a broad range of cognitive enhancements which may generalize beyond the context of video-game play (see sections Hand-Eye Coordination and Reaction Time, Spatial Visualization, Visuospatial Attention, Visual Anticipation and Visual Search Strategies, Temporal Dynamics of Sensory Attention, Exogenous and Endogenous Attention, Task Switching, Fundamental Properties of the Visual System, Visual Perception and Use of Sensory Evidence, Video-Game Play as a Cognitive Intervention). There are, however, concerns about current research practices that need to be addressed (Boot et al., 2011) to clarify the validity of the current results. In addition to the concerns raised by Boot et al. researchers should also monitor gender disparities in their VGP and NVGP samples, seriously assess expertise of their expert VGP sample, and pay closer attention to the genres they possess expertise in and which are used in training studies.

Despite an impressive corpus of findings, research into the practical application of video-game play is restricted to minimizing the effects of cognitive decline in older adults (see section Video-Game Play as a Cognitive Intervention). In addition, all these studies have lacked adequate control conditions making results particularly problematic to interpret. This is particularly surprising as the potential for video-game play to impact on such a broad range of cognitive domains appears to offer many avenues for practical application. Future rigorous research that is capable of determining whether the application of video-game play can stem cognitive decline in older adults is a high priority. Alternatively, researchers could aim to use video-game play as an intervention to ease cognitive deficits in patient populations or further improve cognitive performance in specialist populations.

Early neuroimaging studies (see section Neuroimaging and Video-Game Play) have successfully begun examining the functional neural correlates of enhanced cognitive performance in expert VGPs. So far only motor planning, selective attention, and visual search have been assessed. Future neuroimaging research is needed to assess the functional neural correlates of enhanced cognitive performance. There is also a need to investigate the structural changes that accompany video-game expertise. A longitudinal randomized control training trial that also incorporate neuro-imaging study would be a valuable approach. The recommendations to promote best behavioral research practices are equally applicable to neuroimaging.

The results in section Fundamental Properties of the Visual System, Visual Perception, and Use of Sensory Evidence are particularly exciting as they suggest fundamental elements of the visual system may be heightened by video-game expertise and play. Changes at this level of the visual system potentially underpin a diverse range of downstream performance enhancements presented in this review, such as spatial visualization (section Spatial Visualization), visuospatial attention (section Visuospatial Attention), visual search (section Visual Anticipation and Visual Search Strategies) and temporal order judgments (section Temporal Dynamics of Sensory Attention). Unfortunately, we still have little understanding as to what about video-game play—other than practice—allows it to shape core elements of the visual system. Answering this question may also elucidate why video-game play has the potential to generate such a broad range of generalized enhancements. Understanding the mechanisms that underlie how video-game expertise gives rise to so many enhancements may lead to the efficient and targeted application of video-game play (and other forms of expertise-related interventions) to both education and clinical settings, and more generally permit individuals to make more informed decisions about the potential uses of their spare time.

## Conflict of interest statement

The authors declare that the research was conducted in the absence of any commercial or financial relationships that could be construed as a potential conflict of interest.

## Supplementary material

The Supplementary Material for this article can be found online at: http://www.frontiersin.org/Cognition/10.3389/fpsyg.2013.00629/abstract
