# Supplementary material for: The virtual brain: 30 years of video-game play and cognitive abilities
Source: Front Psychol. 2013 Sep 13;4:629. doi: 10.3389/fpsyg.2013.00629 (PMC3772618; doi:10.3389/fpsyg.2013.00629)
Supplement: Supplementary file 1 [file SupplementaryTable1.pdf]

Supplementary Table 1. Results summary of studies reviewed in the current paper. Studies are organized alphabetically by section they first appear in the paper.

Authors	N	VGP Expertise<br>VGP Training	Tasks	Results	Correlations
**1. Hand-Eye Coordination and Reaction Time**					
Griffith et al. (1983)	31 VGP, 31 NVGP	VGP Expertise:<br>32.97 (27.14) months exp<br>5.74 (10.24) hours per wk	Rotary Pursuit	VGP > NVGP	None
Orosy-Fildes & Allan (1989)	20 NVGP	VGP training:<br>15 minutes	Reaction Time	Post. Train > Pre. Train	N/A
Yuji (1996)	17 VGP, 17 NVGP	VGP Expertise:<br>Enthusiasm towards video-games	Colour and Shape Discrimination	VGP > NVGP	N/A
**2. Spatial Visualization**					
De Lisi & Cammarano (1996)	110 NVGP	VGP training:<br>2, 30 minute sessions over 2 weeks	Mental Rotation	VGP training effect:<br>Post. Train > Pre. Train<br>Female control VGP training effect:<br>Post. Train > Pre. Train	In-game performance/Mental rotation performance (+)
De Lisi & Wolford (2002)	47 NVGP	VGP training:<br>11, 30 minute sessions over 1 month	Mental Rotation	VGP training effect:<br>Post. Train > Pre. Train	N/A
Dorval & Pepin (1986)	70 NVGP	VGP training:<br>40 game plays	Spatial Relations	VGP training effect:<br>Post. Train > Pre. Train	N/A

Gagnon (1985)	58 NVGP	VGP training:<br>5 hours	Spatial Orientation<br>Spatial Visualization<br>Visual Pursuit<br>Hand-eye coordination	VGP training effect: Post. Train > Pre. Train for female spatial visualization only	Prior VGP experience/Visual Pursuit (+), Spatial Visualization (+), Hand-eye coordination (-)
McClurg & Chaillè (1987)	57 VNGP	VGP training:<br>45 minutes, twice a week for 6 weeks	Mental Rotation	Post. Train > Pre. Train	N/A
Okagaki & Frensch (1994)	Exp. 1: 57 NVGP<br>Exp. 2: 53 NVGP	VGP training:<br>12, 30 minute sessions	Mental Rotation<br>Spatial Visualization	VGP training effect:<br>Post. Train > Pre. Train	N/A
Sims & Mayer (2002)	Exp. 1: 53 VGP, 45 NVGP<br>Exp. 2: 24	VGP expertise:<br>>60 lines on Tetris<br>Video-game training:<br>12, 1 hour sessions over 4 weeks	Mental Rotation	VGP > NVGP<br>only for stimuli similar to in-game items	None
Subrahmanyam et al. (1994)	61 NVGP	VGP training:<br>3, 45 minute sessions, maximum 1 week apart	Computerized Spatial Skills Battery	VGP training effect:<br>Post. Train > Pre. Train	N/A
**3. Visuospatial Attention**					
Boot et al. (2008)	Exp. 1: 11 VGP, 10 NVGP<br>Exp. 2: 82 NVGP	VGP expertise:<br>7 hours per week over past 2 years<br>VGP training:<br>15, 1.5 hour sessions over 4-5 weeks	Attentional Blink<br>Corsi Block-Tapping<br>Enumeration<br>Functional Field of View<br>Multiple Object-Tracking<br>Mental Rotation<br>O-Span<br>Ravens Matrices<br>Spatial 2-Back<br>Task Switching<br>Tower of London<br>Visual Shrt. Term Memory	VGP > NVGP for multiple object tracking, mental rotation, task switching and visual shrt. term memory only<br>Control VGP training effect:<br>Post. Train > Pre. Train for mental rotation	N/A

Dye & Bavelier (2010)	Exp. 1: 58 VGP, 103 NVGP<br>Exp. 2: 55 VGP, 99 NVGP<br>Exp. 3: 56 VGP, 95 NVGP	VGP expertise:<br>Self-report playing ‘first’ or ‘third’ person ‘shooters’	Useful Field of View<br>Attentional Blink<br>Multiple Object Tracking	VGP > NVGP, young VGPs display capabilities exceeding or expected of older NVGPs	N/A
Dye et al. (2009)	56 VGP, 75 NVGP	VGP expertise: Self-report playing of any action video-game over the past 6 months	Attentional Network Task	VGP > NVGP across all age ranges	N/A
Green & Bavelier (2003)	Exp. 1: 8 VGP, 8 NVGP<br>Exp. 2: 13 VGP, 13 NVGP<br>Exp. 3: 8 VGP, 8 NVGP<br>Exp. 4: 8 VGP, 8 NVGP<br>Exp. 5: 17 NVGP	VGP expertise:<br>4, 1 hour sessions per day over past 6 months<br>VGP training:<br>1 hour per day over 10 days	Flanker Compatibility<br>Enumeration<br>Useful Field of View<br>Attentional Blink	VGP > NVGP<br>Action VGP training effect:<br>Post. Train > Pre. Train	VGP improvement/task improvement (marginal +)
Green & Bavelier (2006a)	Exp. 1: 8 VGP, 8 NVGP<br>Exp. 2: 8 VGP, 8 NVGP<br>Exp. 3: 32 NVGP	VGP expertise:<br>3-4 days per week over past 6 months<br>VGP training:<br>5 hours per week over 6 weeks	Flanker Compatibility<br>Useful Field of View	VGP > NVGP<br>Action VGP training effect:<br>Post. Train > Pre. Train on useful field of view	None
Green & Bavelier (2006b)	Exp. 1: 13 VGP, 13 NVGP<br>Exp. 2: 17 NVGP<br>Exp. 3: 11 VGP, 11 NVGP<br>Exp. 4: 20 NVGP<br>Exp. 5: 32 NVGP	VGP expertise:<br>3-4 days a week over past 6 months, 5 hours per week over the past 6 months<br>VGP training:<br>10 hours over 15 days, 30 hours over 6 weeks	Enumeration<br>Multiple Object Tracking	VGP > NVGP<br>Action VGP training effect:<br>Post. Train > Pre. Train	N/A
Greenfield et al. (1994)	Exp. 1: 8 VGP, 8 NVGP<br>Exp. 2: 40 NVGP	VGP expertise:<br>>200 000 points on Robot Battle	Divided Attention	VGP > NVGP<br>VGP training effect:<br>Post. Train > Pre. Train	N/A

		Video-game training:<br>5 hours			
Sungur & Boduroglu<br>(2012)	24 VGP, 20 NVGP	VGP expertise:<br>3-4, 1 hour sessions per week over past 6 months	Multiple Identity Tracking<br>Colour Wheel<br>Useful Field of View	VGP > NVGP	N/A
**4. Visual Anticipation and Visual Search Strategies**					
Bialystok<br>(2006)	40 VGP, 57 NVGP	VGP expertise:<br>Self-report	Simon	VGP > NVGP, simple reaction time benefit	N/A
Castel et al.<br>(2005)	Exp. 1: 20 VGP, 20 NVGP<br>Exp. 2: 10 VGP, 10 NVGP	VGP expertise:<br>4, 1 hour sessions per week over past 6 months	Inhibition of Return<br>Visual Search	VGP > NVGP, simple reaction time benefit	N/A
Clark et al.<br>(2011)	15 VGP, 20 NVGP	VGP expertise:<br>6 hours per week over past 6 months	Change Detection	VGP > NVGP	N/A
Hubert-Wallander et al.<br>(2011)	Exp. 1: 10 VGP, 11 NVGP<br>Exp. 2: 19 VGP, 15 NVGP	VGP expertise:<br>5 hours per week over past year	Visual Search<br>Exogenous Cuing	VGP search rate and reaction time > NVGP search rate and reaction time	N/A
Kuhlman & Beitel<br>(1991)	105	N/A	Basin Timer	N/A	VGP experience/Accuracy (+), consistency of anticipation of coincidence (+)
**5. Temporal Dynamics of Sensory Attention**					
Donohue et al.<br>(2010)	18 VGP, 18 NVGP<br>(9 in-between categories)	VGP expertise:<br>2 hours per week over past 6 months	Simultaneity Judgement<br>Temporal Order Judgement	VGP point of subjective simultaneity accuracy > NVGP point of subjective simultaneity accuracy	VGP experience/Subjective simultaneity accuracy (+)

Li et al. (2010)	Exp. 1: 9 VGP, 10 NVGP<br>Exp. 2: 9 VGP, 11 NVGP<br>Exp. 3: 25 NVGP	VGP expertise:<br>5 hours per week over past year<br>VGP training:<br>50 hours over 9 weeks	Backwards Masking	VGP backwards masking effect < NVGP backwards masking effect<br>Action VGP training effect:<br>Post. Train backwards masking effect < Pre. Train backwards masking effect	N/A
West et al. (2008)	Exp. 1: 12 VGP, 12 NVGP<br>Exp. 2: 12 VGP, 12 NVGP	VGP expertise:<br>3-4, 2 hour sessions per week over the past six months	Temporal Order Judgement<br>Signal Detection	VGP point of subjective simultaneity accuracy < NVGP point of subjective simultaneity accuracy, VGP sensitivity > NVGP sensitivity (all except easiest and hardest condition)	N/A
**6. Exogenous and Endogenous Attention**					
Chisholm et al. (2010)	15 VGP, 15 NVGP	VGP expertise:<br>3 hours per week over past 6 months	Attentional Capture	VGP > NVGP, VGP less affected by distractors	N/A
**7. Task Switching**					
Colzato et al. (2010)	17 VGP, 17 NVGP	VGP expertise:<br>4 sessions per week over past 6 months	Task Switching	VGP > NVGP	N/A
Karle et al. (2010)	Exp. 1: 30 VGP, 26 NVGP<br>Exp. 2: 20 VGP, 20 NVGP	VGP expertise:<br>4, 1 hour sessions per week over past 6 months	Task Switching	VGP > NVGP only when proactive interference is low	N/A
Nelson & Strachan (2009)	Exp. 1: 20 NVGP<br>Exp. 2: 20 NVGP	VGP training:<br>1 hour	Location Detection<br>Figure Matching	Action VGP training effect:	N/A

				Post. Train RT > Pre. Train RT<br>Control VGP training effect:<br>Post. Train ACC. > Pre. Train ACC	

8. Fundamental Properties of the Visual System and Use of Sensory Evidence

Buckley et al. (2010)	10 VGP, 10 NVGP	VGP expertise:<br>1 hour per day, at least 4 days per week over past 6 months	Goldmann Kinetic Perimetry	VGP (both central and peripheral) visual field > NVGP	N/A
Green & Bavelier (2007)	Exp. 1: 10 VGP, 10 NVGP<br>Exp. 2: 32 NVGP	VGP expertise:<br>5 hours per week over past 6 months<br>VGP training:<br>30 hours over 6-8 weeks	Visual Crowding	VGP > NVGP (including ‘T’ alone)<br>Action VGP training effect:<br>Post. Train > Pre. Train for crowding only	N/A
Green et al. (2010)	Exp. 1: 11 VGP, 12 NVGP<br>Exp. 2: 11 VGP, 12 NVGP<br>Exp. 3: 25 NVGP<br>Exp. 4: 11 VGP, 12 NVGP	VGP expertise:<br>5 hours per week over past year<br>VGP training:<br>50 hours over 12 weeks	Visual Motion Direction Discrimination<br>Auditory Tone Location Discrimination	VGP sensory accumulation rate > NVGP sensory accumulation rate, VGP evidence threshold < NVGP evidence threshold<br>Action VGP training effect:<br>Post. Train sensory accumulation rate > Pre. Train sensory accumulation rate, Post. Train evidence threshold < Pre. Train evidence threshold	N/A

Li et al. (2009)	Exp. 1: 21 VGP, 19 NVGP<br>Exp. 2: 35 NVGP	VGP expertise:<br>5 hours per week over past 6 months<br>VGP training:<br>50 hours over 5-9 weeks	Contrast Sensitivity	VGP > NVGP<br>Action VGP training effect:<br>Post. Train > Pre. Train	N/A

9. Video-Game Play as a Cognitive Intervention

Basak et al. (2008)	40 NVGP	VGP training:<br>15, 1.5 hour sessions over 5 weeks	Attentional Blink<br>Enumeration<br>Functional Field of View<br>Mental Rotation<br>N-Back<br>O-Span<br>Ravens Matrices<br>Stopping<br>Task Switching<br>Visual Shrt. Term Memory	VGP training effect:<br>Post. Train > Pre. Train for mental rotation, N-back, Ravens matrices, task switching, visual short term memory	N/A
Clark et al. (1987)	14 NVGP	VGP training:<br>14 hours over 7 weeks	Colour Discrimination	VGP training effect:<br>Post. Train > Pre. Train	N/A
Drew & Waters (1986)	15 NVGP	VGP training:<br>1.5 hours, twice a week for 2 months	WAIS<br>Purdue Pegboard<br>Rotary Pursuit	VGP training effect:<br>Post. Train > Pre. Train for all measures	VGP train group/ coordination (+), driving (+), accidents at home (-)
Dustman et al. (1992)	60 NVGP	VGP training:<br>1 hour, 3 times a week over 11 weeks	Visual Sensitivity<br>Symbol Digit Modality<br>Trails B<br>Stroop<br>Finger Tapping<br>Visual Retention<br>Mult. Meas. of Memory<br>Profile of Mood States<br>Sternberg	VGP training effect:<br>Post. Train > Pre. Train for Sternberg only.	N/A

Goldstein et al. (1997)	22 NVGP	VGP training: 5 hours per week over 5 weeks	Sternberg<br>Stroop<br>Emotional Well-Being	VGP training effect: Post. Train > Pre. Train for Stroop and Emotional Well-Being	N/A

10. Neuroimaging and Video-Game Play

Bavelier et al. (2012)	12 VGP, 14 NVGP	VGP expertise: 5 hours per week over past year	Target Detection	VGP > NVGP<br>NVGP Fronto-Parietal Activation > VGP Fronto-Parietal Activation for high load, VGP Visual Area MT < NVGP Visual Area MT for distractors	N/A
Granek et al. (2010)	13 VGP, 13 NVGP	VGP expertise: 12.8 (8.6) hours per week over past 3 years	Visuomotor Transformation	VGP show > frontal activation, NVGP > parietal activation	N/A
Haier et al. (1992)	24 NVGP	VGP training: 30-45 minutes, 5 times a week for 4 to 8 weeks	Tetris	VGP training effect: Whole brain metabolism Post. Train < Pre. Train	VGP improvement/Whole brain metabolism (-)
Mishra et al. (2011)	21 VGP, 20 NVGP	VGP expertise: 9 (2.7) hours per week over past year	Target Detection	VGP > NVGP,<br>VGP SSVEP Amplitude < NVGP SSVEP Amplitude for unattended stimuli,<br>VGP P300 Amplitude > NVGP P300 Amplitude for high load	N/A

11. Challenges to the Video-Game Play Literature

Feng et al. (2007)	Exp. 1: 24 VGP, 24 NVGP<br>Exp. 2: 20 NVGP	VGP expertise: 4 hours per week<br>VGP training: 10 hours over 4 weeks	Useful Field of View	VGP > NVGP<br>Action VGP training effect: Post. Train > Pre. Train,	N/A

				no loss in gains 5 months after training ceased	
Irons et al. (2011)	Exp. 1: 19 VGP, 13 NVGP<br>Exp. 2: 17 VGP, 15 NVGP	VGP expertise:<br>3-4 days per week over previous 6 months	Flanker Compatibility<br>Eriksen Flanker	NVGP positive compatibility at high load/VGP negative compatibility at high load	N/A
Murphy & Spencer (2009)	32 VGP, 29 NVGP	VGP expertise:<br>4, 1 hour sessions per week over past 6 months	Attentional Blink<br>Useful Field of View<br>Inattentional Blindness<br>Repetition Blindness	VGP > NVGP in attentional blink at shortest target interval only	N/A
Quaiser-Pohl et al. (2006)	861	N/A	Mental Rotation	Action VGP in males only, predicts superior mental rotation performance	N/A
Spence et al. (2009)	20 NVGP<br>(10 matched male-female pairs)	VGP training:<br>10 hours over 4 weeks	Attentional Visual Field	Post. Train > Pre. Train, no loss in gains 4 months after training ceased	N/A
